# Burden of antimicrobial prescribing in primary care attributable to sore throat: a retrospective cohort study of patient record data

**DOI:** 10.1186/s12875-024-02371-y

**Published:** 2024-04-17

**Authors:** Kylie S Carville, Niamh Meagher, Yara-Natalie Abo, Jo-Anne Manski-Nankervis, James Fielding, Andrew Steer, Jodie McVernon, David J Price

**Affiliations:** 1 Doherty Epidemiology, Victorian Infectious Diseases Reference Laboratory, at the Peter Doherty Institute for Infection and Immunity, Melbourne, Victoria Australia; 2 Department of Infectious Diseases, The University of Melbourne, at the Peter Doherty Institute for Infection and Immunity, Melbourne, Victoria Australia; 3 Department of Microbiology, Infection Prevention and Control, The Royal Children’s Hospital Melbourne, Melbourne, Victoria Australia; 4 Department of General Practice and Primary Care, The University of Melbourne, Melbourne, Victoria Australia; 5 Centre for International Child Health, Department of Paediatrics, The University of Melbourne, Melbourne, Victoria Australia; 6 Centre for Epidemiology and Biostatistics, Melbourne School of Population and Global Health, The University of Melbourne, Melbourne, Victoria Australia

**Keywords:** Antimicrobial prescribing, Primary care, Sore throat, URTI, *Streptococcus pyogenes*

## Abstract

**Background:**

Reducing antibiotic use in Australia, and the subsequent impact on antimicrobial resistance, requires multiple, sustained approaches with appropriate resources and support. Additional strategies to reduce antibiotic prescribing include effective vaccines, against pathogens such as *Streptococcus pyogenes*, the most common bacterial cause of sore throat. As part of efforts towards assessing the benefits of introducing new strategies to reduce antimicrobial prescribing, we aimed to determine the burden of antimicrobial prescribing for sore throat in general practice.

**Methods:**

General practice activity data from 2013 – 2017 derived from the first 8 practices participating in the ‘Primary Care Audit, Teaching and Research Open Network’ (Patron) program were analysed according to reason for visit (upper respiratory tract infection, URTI, or sore throat) and antibiotic prescription. The main outcome measures were percentage of sore throat or URTI presentations with antibiotic prescription by age.

**Results:**

A total of 722,339 visits to general practice were made by 65,449 patients; 5.7% of visits were for URTI with 0.8% meeting the more specific criteria for sore throat. 66.1% of sore throat visits and 36.2% of URTI visits resulted in antibiotic prescription. Penicillin, the recommended antibiotic for sore throat when indicated, was the antibiotic of choice in only 52.9% of sore throat cases prescribed antibiotics. Broader spectrum antibiotics were prescribed more frequently in older age groups.

**Conclusions:**

Frequency of antibiotic prescribing for sore throat is high and broad, despite Australian Therapeutic guideline recommendations. Multiple, sustained interventions to reduce prescribing, including availability of effective *S. pyogenes* vaccines that could reduce the incidence of streptococcal pharyngitis, could obviate the need to prescribe antibiotics and support ongoing efforts to promote antimicrobial stewardship.

**Supplementary Information:**

The online version contains supplementary material available at 10.1186/s12875-024-02371-y.

## Background

Control of *Streptococcus pyogenes* (Strep A) diseases, that directly affect >750 million people and cause more than 500,000 deaths each year [1], is a high priority. Strep A has a disproportionately high burden among the First Nations peoples of Australia and Canada, the Pacific region, sub-Saharan Africa, south central Asia, and settings of disadvantage worldwide [2, 3]. However, a significant burden of disease is distributed across all settings and life stages [4, 5]. Infections range from common superficial skin infections (>150 million prevalent cases globally) and pharyngitis (sore throat, >600 million/year), to life-threatening invasive disease (>600,000/year) with a considerable surge in incidence of invasive Strep A noted in USA, Europe and Australia after pandemic years 2020-2021 [6, 7]. Post-infectious complications include acute rheumatic fever (ARF) leading to rheumatic heart disease (RHD, prevalence ~34 million), and glomerulonephritis [8]. A vaccine against Strep A would reduce the varied burden of disease, and the World Health Organization (WHO) has prioritised Strep A as a priority pathogen for accelerated vaccine development [9]. A key factor is establishing an economic case considering the full scope of costs and benefits, including the potential to reduce the secondary burden of antimicrobial prescribing for suspected Strep A infection, and the consequent contribution to antibiotic resistance.

In Australia, the burden of Strep A disease is distributed across every level of the health system. Direct and indirect costs associated with common but less severe syndromes such as sore throat manifest through primary care and hospital visits, work and school absences, antibiotic use and misuse, leading to antimicrobial resistance [10, 11]. Whilst Strep A disease most commonly manifests as pharyngitis, sore throat is most often attributable to viral pathogens. Strep A causes an estimated 10–20% of sore throat cases, and is usually self-limiting [12]. It is difficult to clinically distinguish Streptococcal pharyngitis from viral causes of sore throat, although the latter is more likely to be associated with upper respiratory tract infection (URTI) symptoms such as cough, hoarse voice, and nasal congestion. A risk factor-based approach to prescribing antibiotics for suspected streptococcal pharyngitis and tonsillitis is advised in the Australian Therapeutic Guidelines [13]. Antibiotic prescription is recommended only for patients 2 to 25 years from populations with high incidence of rheumatic fever, and patients with existing rheumatic heart disease, scarlet fever, or very severe symptoms including severe throat pain, dysphagia or need for hospitalisation. Despite these recommendations and national efforts targeted at reducing antibiotic prescriptions, several studies have shown high incidence of antibiotic prescribing for URTI and sore throat [14–16]. Treatment guidelines for sore throat vary substantially across countries and regions [17] with a lower threshold for investigation and treatment of Strep A pharyngitis recommended in some countries such as the USA [18], further increasing the global burden of antimicrobial prescribing.

This study measured the relative frequency of URTI, sore throat and pharyngitis presentations among different age groups in a sample of general practice (GP) clinics in the state of Victoria (population 6,500,000), and frequency and type of antibiotic prescribed, to quantify antimicrobial prescribing and potential vaccine benefits.

## Methods

### Extraction of patient data

URTI presentations to the first eight GP clinics participating in the ‘Primary Care Audit, Teaching and Research Open Network’ (Patron) program were retrospectively reviewed. Patron is a research initiative of The University of Melbourne’s Department of General Practice Data for Decisions Program, that aims to make better use of existing primary care data to improve knowledge, medical education, policy, and the way medical care is delivered [19].

Data extraction was performed by the Patron team with data de-identified at the source of extraction. Data were extracted for patients who had a current medical record (i.e., were not marked inactive as a result of death, moving clinics, or not attending for a set period of time) at the time of data extraction (May 2019) and who had visited one of the eight practices over a five-year period, 2013 to 2017. All visit records were selected for extraction, including clinical and administrative records. Non-clinical records were subsequently excluded if they were interactions that were: 1) not conducted by a GP, or; 2) unless specifically recorded as telehealth, occurred via telephone, email, or SMS, or were listed as a non-visit. The reason for visit field was only extracted if terms associated with URTI were listed as the reason for presentation. Reasons which were not associated with URTI were not extracted, and so in these instances the reason for visit field was blank. All prescription records from 2013 and 2017 were extracted separately and linked to visit records using visit ID or date.

### Coding of variables of interest

Reason for visit was categorised into three levels with increasing specificity for sore throat caused by Strep A.

#### URTI

The free text terms in the reason for visit variable used to extract potential URTI presentations are listed in Additional file 1. These terms were broad in nature in order to capture as many relevant records as possible and included those used for sore throat (as defined below), variations on the term URTI and pathogen/disease names that cause URTI. Extracted records were then reviewed in consultation with clinical experts to determine a shortlist of exclusion terms (Additional file 1), which were applied to reduce misclassification of visits as being related to URTI.

#### Sore throat

Any visit reason including the terms “sore throat”, “throat infection”, “tonsillitis”, “pharyngitis”, “epiglottitis”, “tonsillar abscess”, “strep” or “scarlet fever”.

#### Streptococcal pharyngitis

Any visit reason including the terms “strep” or “scarlet fever”.

#### Antibiotic prescriptions

Antibiotic prescriptions associated with a clinical visit were identified in prescription records. Coding for any antibiotic was defined using the Anatomical Therapeutic Chemical (ATC) and Australian Medicines Terminology (AMT) codes obtained from publicly available data on the Pharmaceutical Benefits Scheme (PBS) website [20]. Antibiotics that may be prescribed for sore throat in practice were identified from the Australian Therapeutic Guidelines and with advice of experts (Additional file 2). Recommended antibiotics for sore throat according to the Australian Therapeutic Guidelines are: penicillin (including phenoxymethylpenicillin, benzathine benzylpenicillin, procaine benzylpenicillin) as first line, or cefalexin or azithromycin depending on degree of penicillin allergy [13]. Amoxicillin is also listed as a recommended antibiotic in the Infectious Disease Society of America guideline on management of Streptococcal pharyngitis [21] or an alternative antibiotic in the Australian Therapeutic Guidelines in cases where children are unable to tolerate the liquid formulation of phenoxymethylpenicillin. Antibiotics are not generally recommended for URTI.

### Data analysis

Patient demographics, visit patterns and patient numbers were summarised, overall and by clinic. The overall burden of URTI, sore throat, and pharyngitis was quantified by the number and proportion of clinical visits, and the number and proportion of patients presenting at least once with these conditions of interest. These data were also examined by age group, sex, season, clinic, and year.

Prescription of antibiotics was summarised as the frequency and proportion of URTI and sore throat presentation for which any antibiotic was prescribed. Among clinical visits associated with an antibiotic prescription, the frequency and proportion that received: 1) any Australian Therapeutic Guidelines recommended antibiotic; 2) each individual Australian Therapeutic Guidelines recommended antibiotic, and; 3) each commonly prescribed non-recommended antibiotic. Antibiotic prescribing practices were also examined by age group, sex, season, clinic, and year.

#### Repeat presentations

Repeat presentations for the same episode of disease were defined as the overall number and proportion of visits for URTI or sore throat that occurred within a pre-specified timeframe, defined as either within 1) 7 days, or; 2) 28 days of another visit for the same presentation. Repeat visits may represent cases of chronic sore throat, which in turn could contribute to increased antibiotic prescription in this cohort. To understand the impact of repeat presentations, the frequency and proportion of Australian Therapeutic Guidelines recommended and non-recommended antibiotic prescribing at repeat visits for sore throat or URTI was reported.

Data cleaning, linkage of data tables, analysis and creation of figures was performed using Stata version 16.0 and R version 4.2.2 [22, 23].

## Results

### Presentations

From 2013 to 2017 a total of 722,339 clinical visits were made across the eight practices by 65,449 patients in the cohort, with an average of five visits per patient (interquartile range [IQR]: 2, 13). Most (714,755, 99.0%) visits were recorded as occurring in the GP clinic, with other visits occurring at other locations such as aged care facilities or homes. The median age of patients was 32 years (IQR: 20, 48) and 55.0% were female (35,991), with similar age and sex distributions across the clinics apart from one clinic with majority male presentations (Table 1). Six clinics were metropolitan and two were regional.

**Table 1:** Clinic characteristics including numbers of presentations and patients 2013 to 2017; and age and sex distribution of patients

	**Clinic 1**	**Clinic 2**	**Clinic 3**	**Clinic 4**	**Clinic 5**	**Clinic 6**	**Clinic 7**	**Clinic 8**
Total visits	78,734	64,368	30,611	44,084	210,953	74,921	116,649	102,019
Average total visits per year	15,747	12,874	6,122	8,817	42,191	14,984	23,330	20,404
Total unique patients	4,155	8,599	9,049	4,837	13,034	9,856	8,389	7,530
Average visit number per patient, 2013–2017 (IQR)	10 (4, 23)	4 (2, 10)	2 (1, 4)	1 (1, 4)	8 (3, 19)	4 (2, 10)	6 (2, 18)	8 (3, 17)
***Patient characteristics***								
***Sex***^***a***^								
Female	2,305 (55.5%)	4,437 (51.6%)	5,524 (61.0%)	1,142 (23.6%)	7,890 (60.5%)	5,529 (56.1%)	4,705 (56.1%)	4,459 (59.2%)
Male	1,845 (44.4%)	4,161 (48.4%)	3,475 (38.4%)	3,653 (75.5%)	5,140 (39.4%)	4,326 (43.9%)	3,678 (43.8%)	3,066 (40.7%)
Age^b^ (years) (IQR)	41 (24, 57)	30 (12, 42)	30 (25, 37)	38 (26, 51)	32 (18, 50)	32 (16, 48)	32 (18, 52)	36 (21, 51)

^a^114 patients had other values recorded for sex (104 missing, 7 other, 3 unknown)

^b^26 patients were missing age

Over the five years, 5.7% of all visits had URTI recorded as the reason for visit, with 0.8% of all visits recording the more specific terms for sore throat (Table 2). There were very few presentations coded as streptococcal pharyngitis (*n*=73) — the majority of these were recorded in 2017 (*n*=59) from two clinics. Therefore, the category of streptococcal pharyngitis is not further described. Just under 7% of patients had ever visited a clinic for sore throat over 2013–2017, and 30% of patients for URTI (Table 2).

**Table 2:** Number of presentations, and the number of patients presenting at least once, with upper respiratory tract infection (URTI) and sore throat in the five-year period from 2013 to 2017, overall and by sex and age group

	**URTI, no. (%)**	**Sore Throat, no. (%)**
**Total** (722,339 visits by 65,449 patients)		
Number of clinical presentations	41,532 (5.8%)	5,862 (0.8%)
Patients presenting at least once	19,746 (30.2%)	4,330 (6.6%)
**Patient characteristics**		
**Sex**^**a**^		
** Female** (444,325 visits by 35,991 patients)		
Number of clinical presentations	24,811 (5.6%)	3,596 (0.8%)
Patients presenting at least once	11,496 (31.9%)	2,627 (7.3%)
** Male** (277,764 visits by 29,344 patients)		
Number of clinical presentations	16,711 (6.0%)	2,264 (0.8%)
Patients presenting at least once	8,241 (28.1%)	1,701 (5.8%)
**Age group**^**b**^		
** 0 – 4 years** (50,968 visits by 6,942 patients)		
Number of clinical presentations	8,892 (17.4%)	651 (1.3%)
Patients presenting at least once	3,474 (50.0%)	484 (7.0%)
** 5 – 14 years** (42,920 visits by 6,968 patients)		
Number of clinical presentations	5,598 (13.0%)	1,090 (2.5%)
Patients presenting at least once	2,777 (39.9%)	787 (11.29%)
** 15 – 49 years** (307,326 visits by 39,405 patients)		
Number of clinical presentations	18,129 (5.9%)	3,500 (1.1%)
Patients presenting at least once	9,758 (24.8%)	2,599 (6.6%)
** 50+ years** (321,094 visits by 15,721 patients)		
Number of clinical presentations	8,912 (2.8%)	620 (0.2%)
Patients presenting at least once	4,382 (27.9%)	512 (3.3%)

^a^114 patients are not included in the sex subgroup (104 missing, 7 recorded as “other”, 3 recorded as “unknown”)

^b^26 patients are not included in the age subgroup due to missing age records. Note that patients may be counted in two different age categories over the course of the study

The yearly proportion of presentations recorded as URTI and sore throat remained constant. Across years, a median of 19.7% (IQR: 18.1%, 19.9%) of patients ever presented with an URTI; 3.4% (IQR: 2.8%, 3.7%) with sore throat. There was little difference in URTI or sore throat presentations amongst males and females over the five years (Table 2).

**Fig. 1:**
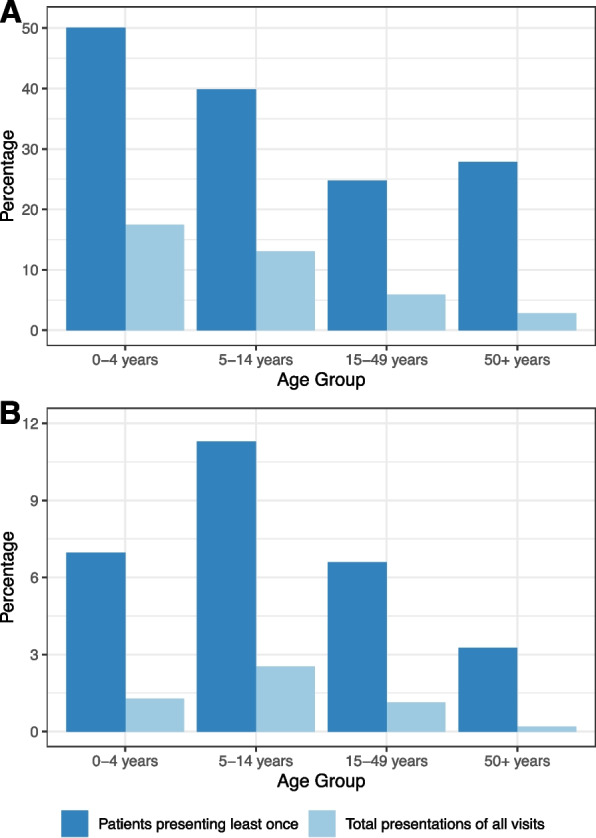
Total visits presenting with, and patients who presented at least once with **A** upper respiratory tract infection (URTI) and **B** sore throat, from 2013–2017 by age group.

Sore throat presentations were most common in school-aged children, with 11.3% of those aged 5 to 14 years presenting at least once for sore throat (Fig. 1). Visits for URTI were highest amongst those aged under 5 years, accounting for 17.4% of all visits in this age group (Fig. 1). The median proportion of children under 5 years who presented for URTI at least once in a year was 39.6% (IQR: 37.9%, 39.8%). Presentations peaked in winter, when 0.9% of all presentations were for sore throat and 7.8% for all URTI (compared with 0.7% and 3.7%, respectively, in summer months).

### Prescriptions

In total, there were 544,343 records of prescriptions, of which 491,086 (90.2%) were able to be linked to a clinical visit. Unmatched prescriptions were largely linked to non-clinical entries in patient records, such as phone calls to administrative staff, of which only 565 and 107 were related to URTI or sore throat, respectively. Of the linked prescriptions, 83,938 (17.1%) were for antibiotics. Overall, 11.6% of all GP visits were associated with an antibiotic prescription (Table 3). URTI was recorded as the reason for the visit in 15,057 (17.9%) of presentations where antibiotics were prescribed. There were 3,876 antibiotic prescriptions for the more specific category of sore throat, which accounted for 4.6% of all antibiotic prescriptions.

**Table 3:** Antibiotic prescribing practices in the five-year period from 2013 to 2017, overall and for upper respiratory tract infection (URTI) and sore throat

	**URTI, *N* (%)**	**Sore Throat, *N* (%)**	**Total Visits, *N* (%)**
**Total presentations**	41,532	5,862	722,339
**Prescribed any antibiotic**	**15,057 (36.3%)**	**3,876 (66.1%)**	**83,938 (11.6%)**
**Therapeutic Guidelines recommended antibiotics**			
Total	2,834 (18.8%)	2,222 (57.3%)	17,037 (20.3%)
Phenoxymethylpenicillin	1,833 (12.2%)	1,685 (43.5%)	2,581 (3.1%)
Benzylpenicillin	376 (2.5%)	367 (9.5%)	532 (0.6%)
Cefalexin	571 (3.8%)	157 (4.1%)	12,281 (14.6%)
Azithromycin	54 (0.4%)	13 (0.3%)	1,643 (2.0%)
**Key antibiotics prescribed but not recommended by Therapeutic Guidelines**			
Amoxicillin	5,944 (39.5%)	897 (23.1%)	18,111 (21.6%)
Amoxicillin clavulanic acid	1,661 (11.0%)	179 (4.6%)	7,621 (9.1%)
Roxithromycin	1,079 (7.2%)	108 (2.8%)	3,501 (4.2 %)
Cefaclor	254 (1.7%)	22 (0.6%)	1,051 (1.3%)
Doxycycline	97 (0.6%)	1 (0.03%)	962 (1.1%)

The denominator for “any antibiotic” percentage is total number of presentations; the denominator for specific antibiotic percentages is the number prescribed any antibiotic

Two thirds (66.1%) of patients presenting with sore throat were prescribed antibiotics, compared to 36.2% of patients presenting with URTI. Of the antibiotics prescribed for sore throat, the most common was penicillin (52.9%; phenoxymethylpenicillin 43.5% and benzylpenicillin 9.5%), followed by amoxicillin (23.1%). Over half (57.3%) of the prescriptions were for antibiotics recommended in the Australian guidelines. Of the antibiotic prescriptions for URTI, amoxicillin was prescribed most commonly (39.5%), with less use of penicillin (14.7%).

**Fig. 2:**
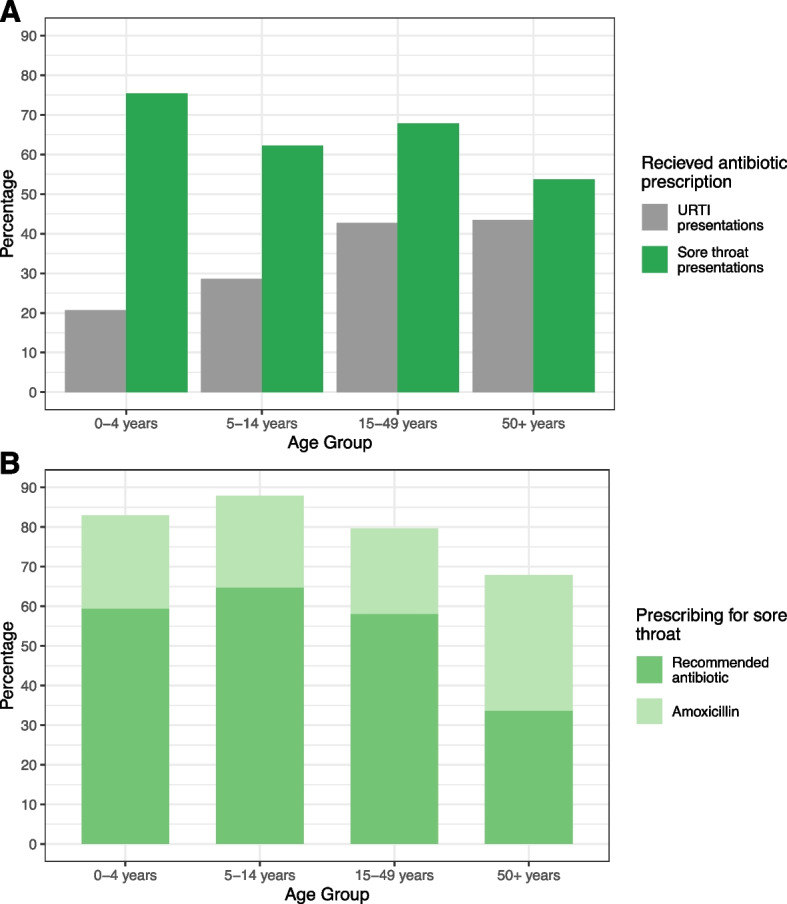
**A** Proportion of presentations for sore throat and upper respiratory tract infections (URTI) from 2013–2017 which received an antibiotic prescription, by age group; **B** Proportion of antibiotic prescriptions for sore throat from 2013–2017 which were recommended antibiotics or amoxicillin, by age group.

Antibiotic prescription for sore throat did not differ according to sex and was highest in children aged under 5 years, for whom 75% of presentations for sore throat resulted in antibiotic prescription (Fig. 2). Penicillin was the most commonly prescribed antibiotic in most age groups: 56.2% (276/491) of children under 5 years and 60.6% (411/678) of children aged 5 to 14 years who received an antibiotic were prescribed penicillin, along with 53.6% (1,272/2,374) of those 15 to 49 years, but only 27.9% (93/333) of those aged over 50 years. Those aged over 50 years who received an antibiotic were prescribed amoxicillin in 114 (34.2%) of 333 cases compared with approximately 20% of younger age groups, and received more amoxicillin clavulanic acid and roxithromycin (8.7% and 8.4% of prescriptions respectively, compared with 2% or less in children under 15 years) (Fig. 2).

The proportion of visits where an antibiotic was prescribed for an URTI increased with increasing age, doubling from 20.7% among children aged less than 5 years to over 40% for those aged over 15 years (Fig. 2). The majority of prescribing for children aged under 5 years with an URTI was amoxicillin (55.5%, 1,020/1,839). People aged over 50 years with an URTI mostly received amoxicillin (34.8%, 1,347/3,874), amoxicillin clavulanic acid (14.4%, 559/3874), and roxithromycin (12.0%, 466/3,874).

Prescription of any antibiotic was variable across different clinics regardless of location. For sore throat presentations, 57.5% to 81.5% of visits received any antibiotic. For URTI presentations it was 27.6% to 44.3% of visits. Of the two clinics prescribing the most antibiotics for sore throat (both over 80%), one prescribed the lowest proportion of recommended antibiotics (16.4%; 14.6% penicillin), while the other prescribed a higher proportion (64.4%; 58.3% penicillin) consistent with most other clinics, which ranged between 47.3% and 67.5%. Prescription of penicillin was relatively constant over the five-year period of the study, however, prescription of amoxicillin increased from 12.8% of all prescriptions for sore throat in 2013 to 24.6% in 2017.

### Repeat presentations

Of all clinical visits for sore throat, there were 283 and 440 instances of multiple presentations for the same condition within 7- and 28-day timeframes, respectively (Table 4.). This included a total of 308 (5.3%) visits in 7 days and 518 (8.8%) visits in 28 days. For URTI, there were 2,533 episodes of multiple visits within 7 days and 4,449 episodes of multiple visits within 28-days. The majority of repeat presentations for sore throat were limited to two visits (7 days: two visits were 91.5% of repeat visits [*n*=259]; 28 days: two visits were 85.2% of repeat visits [*n*=375]). The proportion of repeat presentations limited to two visits was similar for URTI (7 days: 90.1% [*n*=2,283], and 81.7% [*n*=3,636]).

**Table 4.** Repeat visits and antibiotic prescribing for upper respiratory tract infection (URTI) or sore throat within 1) 7 days, or; 2) 28 days of a prior presentation, overall and by visit number. Data are inclusive of all records in the five-year period from 2013 to 2017.

	**URTI** ***N* = 41,532**		**Sore Throat** ***N* = 5,862**	
	**7-day, *N* (%)**	**28-day, *N* (%)**	**7-day, *N* (%)**	**28-day, *N* (%)**
**Total episodes of repeat presentations**	**2,533**	**4,449**	**283**	**440**
**Received ≥1 antibiotic prescription(s)**	**1,516 (59.8%)**	**2,743 (61.7%)**	**245 (86.6%)**	**385 (87.5%)**
Any antibiotic prescribed at first visit	839 (33.1%)	1,629 (36.6%)	196 (69.3%)	319 (72.5%)
Any antibiotic prescribed at second visit	903 (35.6%)	1,631 (36.7%)	106 (37.5%)	193 (43.9%)
Any antibiotic prescribed at third and subsequent visit(s)^a^	96/293 (32.8%)	372/1,098 (33.9%)	7/25 (28.0%)	35/78 (44.9%)
**Received ≥1 Australian Therapeutic Guidelines recommended antibiotic(s)**	**326 (12.9%)**	**565 (12.7%)**	**177 (62.5%)**	**261 (59.3%)**
Recommended antibiotic at first visit	200 (7.9%)	341 (7.7%)	138 (48.8%)	205 (46.6%)
Recommended antibiotic at second visit	135 (5.3%)	241 (5.4%)	53 (18.7%)	96 (21.8%)
Recommended antibiotic at third or subsequent visit(s)^a^	14/293 (4.8%)	53/1,098 (4.8%)	3/25 (12.0%)	15/78 (19.2%)

^a^The denominator is provided for third and subsequent visits as the total number of repeat presentations was variable

**Fig. 3:**
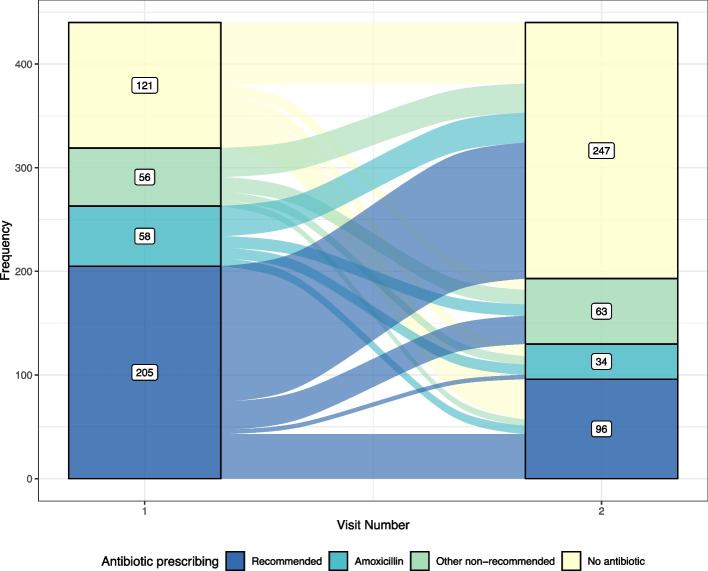
Alluvial plot of antibiotic prescribing outcomes of the first two repeat visits for 440 episodes of sore throat within 28 days of first presentation from 2013–2017. Patients were categorised by whether they received: i) an antibiotic recommended for treatment of sore throat, as indicated by Australian Therapeutic Guidelines; ii) amoxicillin; iii) any other non-recommended antibiotic, or; iv) no antibiotic prescription.

Antibiotics were prescribed at least once for 86.6% and 87.5% of all episodes of multiple visits for sore throat within 7 and 28 days, respectively. The majority of these repeat visits received at least one Australian Therapeutic Guidelines recommended antibiotic (62.5% for the 7-day timeframe and 59.3% for the 28-day timeframe). The pattern of antibiotic prescription for the first two visits for sore throat within 28 days is also characterised in Fig. 3. At each visit, patients were characterised as receiving: i) an antibiotic recommended for treatment of sore throat, as indicated by Australian Therapeutic Guidelines; ii) amoxicillin; iii) any other non-recommended antibiotic, or; iv) no antibiotic prescription. Antibiotic prescribing was higher at the first visit (319 [72.5%]) than the second visit (193 [43.9%]). Prescription rates did not change from the second visit when considering subsequent repeat visits for sore throat (35/78 [44.9%]). Recommended antibiotics were also more common at the first visit compared to the second, comprising 205 (64.3%) and 96 (49.7%) of all prescriptions at the first and second visits respectively.

## Discussion

We reviewed a large dataset of 722,339 clinical visits to eight GP practices in Victoria over a five-year period to determine frequency of antibiotic prescriptions for sore throat and URTI. Antibiotics were prescribed in 66.1% of cases of sore throat, and 36.2% cases of URTI. Penicillin, the antibiotic of choice where indicated, represented 53% of antibiotic prescriptions for sore throat. Even in the setting of suspected bacterial pharyngitis, antibiotics are only recommended in select cases [13]. Yearly national surveys of general practice activity have estimated that 19% to 40% of tonsillitis/pharyngitis cases may require antibiotic treatment based on Australian Therapeutic Guidelines criteria for antibiotic prescription [15].

Presentations recorded as sore throat were most common among those aged 5 to 15 years, consistent with the known peak incidence of streptococcal pharyngitis occurring in school aged children [3]. A bacterial cause is less likely in adults: Strep A is cultured in 15% to 36% of children compared to 5% to 17% of adults with sore throat [12]. Despite this, little difference was seen in prescribing in 5 to 15 year-olds compared with those less than 5 years and 15 to 49 years of age. Furthermore, there was substantial antibiotic prescribing for sore throat in those aged 50 years or older in this data (53.7%). A cohort study of GP trainees in 2010 – 2012 reported less antibiotic prescribing for sore throat for those aged over 50 years (7.4%). [16] This could in part be due to differences in prescribing practices by seniority [24], which could not be assessed in this study as GP level data was not available, although it is likely a range of career stages are represented in our data. These contrasting results highlight the diversity of antibiotic prescribing practices in primary care. These differences may be attributable to the specific patient populations served by clinics included or the differences in the methods used to quantify antibiotic prescribing in either study.

Although this study found high levels of antibiotic prescribing overall, antibiotic type was more likely to follow the Australian Therapeutic Guidelines for children than older adults. People aged over 50 years were more likely to be prescribed broader spectrum antibiotics for sore throat, with amoxicillin prescribed for 34.2% of sore throat presentations in those over 50 compared to 21.5% to 23.4% in the younger age groups. Older age groups, aged 15 to 49 and over 50 years, were more likely to receive antibiotics for URTI (42.7% and 43.5% of presentations, respectively) relative to children (20.7% of presentations in those aged under 5 and 28.6% of presentations in the 5 to 14 age group). Only 32% of those aged over 50 years received an Australian Therapeutic Guideline recommended antibiotic for sore throat. Another smaller study using Patron data showed 440/795 (55.3%) prescriptions for pharyngitis or tonsillitis in individuals aged 12 years or older were recommended by the Australian Therapeutic Guidelines, noting that this study used more specific diagnostic terms. [25]

The antibiotic prescription rate for sore throat and URTI from 2013 – 2017 in our study is consistent with previous Australian studies, where antibiotics were prescribed for 21.6% of URTI presentations (2010 – 2012) [16], and 71.5% of sore throat presentations (2010 – 2014) [14], but not as high as for the more specific diagnosis of acute pharyngitis or tonsillitis (94%, 2010 – 2015) [15]. It is possible that current antibiotic prescribing practices have changed since 2017, however, high antibiotic prescribing in primary care continues to be reported nationally [26]. Repeat presentations for sore throat do not appear to be the driver of increased antibiotic prescribing observed in this study: only 5.3% of visits were repeat visits within a 7 day window, and; among repeat clinical visits for sore throat, antibiotics were prescribed at a higher rate at the first visit (72.5%), than at the second (43.9%) or subsequent (44.9%) visits for sore throat, potentially indicating that patients with repeat visits had more severe illnesses at the outset.

There are limitations in this analysis. The “Reason for visit” field is not always completed in electronic medical records, and may not include all reasons why a patient presented, thus URTI and sore throat presentations may be an underestimate. Moreover, attribution of prescribing to any given presenting symptom or syndrome is limited to inference in the absence of a stated indication, and we could not identify where amoxicillin was prescribed in young children for improved tolerability. The cohort comprised of patients with a current medical record at the time of record extraction (May 2019), thus patients who may have attended these clinics earlier in the study period, but were not active in May 2019, were not included. This skews the data towards more recent presentations; however, this is not expected to be a notable issue as presentations for sore throat and URTI were constant across the time, and similar to other data sources. The Patron network was in its infancy at the time this study was initiated, and only a small number of clinics were available for inclusion. These clinics may not be representative of broader prescribing practices across primary care settings. Finally, the Australian Therapeutic Guidelines is one of multiple published criteria available in Australia [27]. While widely used, access to these guidelines is subscription-based and so not universally available to Australian GPs. For example, any GPs using the Infectious Disease Society of America guidelines may be prescribing amoxicillin [21], which may explain the high use of this antibiotic in our cohort. In addition, more specific guidelines exist which are more applicable for remote areas and/or Aboriginal and Torres Strait Islander peoples.

Reasons for antibiotic prescription in primary care are multifactorial, including fear of rare sequelae [28]. Lowering antibiotic prescribing rates is challenging. Our study did not include data regarding specific reasons for prescription, or sufficient details to understand risk factor profiles that may lead to increased prescribing under Australian guidelines, such as for ARF/RHD, although the prescribing rate was consistent across urban and regional clinics included in this cohort, and the overall annual incidence of ARF and RHD in Victoria is low [29]. The data also did not capture the performance of or results of investigations (e.g., throat culture). Australian guidelines do not place emphasis on performing investigations for sore throat and rapid point-of-care tests are not widely available, with throat culture potentially performed in 18 – 37% of cases [30]. Even when available, rapid point-of-care testing was only associated with a 16% absolute reduction in the rate of antibiotic prescribing for sore throat in children in the United States [31]. A Cochrane review of interventions to improve antibiotic prescribing in ambulatory care found multi‐faceted interventions combining physician, patient and public education were most successful, but only one of four studies demonstrated a sustained reduction in the incidence of antibiotic‐resistant bacteria [32]. Such interventions are costly and resource-intensive: one intervention in 56 GP practices in Australia reduced antibiotic prescriptions by only 7% [33], consistent with other reports of suboptimal or unsustained reductions as a result of education or antimicrobial stewardship interventions [31, 34, 35].

## Conclusions

This study has demonstrated high, often broad-spectrum, antibiotic prescribing for sore throat in a large sample of GP visits. Reports of suboptimal reductions in unnecessary antibiotic prescribing as a result of costly, multi-faceted interventions suggests that additional strategies are required, such as effective vaccines, alongside ongoing GP and community education and clinical decision support tools. Further work to understand the drivers for antibiotic prescribing is crucial to determine how resources can best be allocated to the wide spectrum of interventions aimed at improving antimicrobial stewardship in the GP clinic. Reducing the demonstrated burden of prescribing for sore throat and URTI through a multifaceted approach that includes vaccination could help combat the ongoing global health threat of antimicrobial resistance [31].

### Supplementary Information

**Supplementary Material 1.**

**Supplementary Material 2.**

## Data Availability

The data that support the findings of this study are available from Patron but restrictions apply to the availability of these data, which were used under agreement for the current study, and so are not publicly available. Authors had full access to the data (including statistical reports and tables).

## Abbreviations

AMT: Australian Medicines Terminology
ARF: Acute rheumatic fever
ATC: Anatomical Therapeutic Chemical
GP: General practice
IQR: Interquartile range
Patron: Primary Care Audit, Teaching and Research Open Network
RHD: Rheumatic heart disease
PBS: Pharmaceutical Benefits Scheme
URTI: Upper respiratory tract infection
WHO: World Health Organization
