# Supplementary material for: Burden of antimicrobial prescribing in primary care attributable to sore throat: a retrospective cohort study of patient record data
Source: BMC Prim Care. 2024 Apr 17;25:117. doi: 10.1186/s12875-024-02371-y (PMC11022400; doi:10.1186/s12875-024-02371-y)
Supplement: Supplementary file 1 — Supplementary Material 1. [file 12875_2024_2371_MOESM1_ESM.docx]

# Additional file 1

List of terms used to filter the ‘Reason for visit’ field for URTI presentations during the extraction of clinic data. These terms were derived with clinical input and were broad in nature in order to capture as many relevant records as possible. If the ‘Reason for visit’ field contained at least one of these terms, the record was selected for extraction.

- Abscess – Tonsillar
- Acid fast bacilli – microculture
- Acid fast bacilli – microculture – nasal scraping
- Acute epiglottitis
- Acute pharyngitis
- Acute sinusitis
- Adenovirus infection
- Adenovirus serology
- Airway Blockage
- Bacterial tonsillitis
- Bird Flu – H5N1 infection
- Blocked nose
- Bordetella
- Bordetella Infection
- Bordetella pertussis – M&C
- Bordetella pertussis infection
- Chronic Sinusitis
- Chronic Tonsillitis
- Cold – Common
- Common cold
- Corynebacterium diphtheriae M & C
- Corynebacterium diptheriae infection
- Coryza
- Cough
- Cough – Blood
- Cough – Nocturnal
- Cough – post infective
- Cough – post viral
- Cough – Swallowing
- Coxsackie A – serology
- Coxsackie B – serology
- Coxsackie Virus A Infection
- Coxsackie virus infection
- Croup
- Diphtheria
- Discharge from nose
- EBV infection
- Epiglottitis
- Epstein Barr virus infection
- Epstein Barr virus serology
- Follicular tonsillitis
- Herpangina
- Human Swine Influenza infection
- Infection - Bordetella pertussis
- Infection - Haemophilus influenzae
- Infection - Influenza virus
- Infection - Parainfluenza
- Infection - Parainfluenza 1 Virus
- Infection - Parainfluenza 2 Virus
- Infection - Parainfluenza 3 Virus
- Infection - Tonsil
- Infectious mononucleosis
- Infectious mononucleosis - serology
- Inflammation - Labyrinth
- Inflammation - Larynx
- Inflammation - Mastoid
- Inflammation - Mouth
- Inflammation - Nasal mucosa
- Inflammation - Pharynx
- Influenza A infection
- Influenza B infection
- Influenza H1N1
- Influenza infection
- Influenza like illness
- Influenza serology
- Inspiratory wheeze
- Labyrinthitis
- Laryngitis
- Laryngotracheobronchitis
- Loss of smell
- Lost voice
- M&C - Bordetella pertussis
- M&C - Corynebacterium diptheriae
- M&C - Haemophilus influenzae
- Membranous pharyngitis
- Membranous tonsillitis
- Microculture - Nasal scraping
- Microculture - Sputum
- Moxarella infection
- Myringitis - viral
- Nasal blockage
- Nasal congestion
- Nasal discharge
- Nasal mucosa lesion
- Nasal mucosal inflammation
- Pain - swallowing
- Pain - throat
- Parainfluenza Infection
- Parainfluenza Type 1 Infection
- Parainfluenza Type 2 Infection
- Parainfluenza Type 3 Infection
- Pertussis infection
- Pharyngeal Swab
- Pharyngitis
- Pharyngitis - infective
- Pig Flu - H1N1 infection
- Post Bronchitis Cough
- Post viral cough
- Post-viral Cough
- Recurrent sinusitis
- Recurrent tonsillitis
- Recurrent upper respiratory tract infection
- Respiratory syncitial virus infection
- Respiratory Tract Infection
- Rhinitis
- Rhinitis - Rebound
- Rhinoconjunctivitis
- RSV
- RSV infection
- RTI - viral
- RTI (Respiratory Tract Infection)
- Runny nose
- Scarlet fever
- Sino-bronchitis - acute
- Sino-bronchitis - chronic
- Sinusitis
- Sinusitis - Acute
- Sinusitis - Chronic
- Sinusitis - ethmoid
- Sinusitis - frontal
- Sinusitis - maxillary
- Sinusitis - recurrent
- Sinusitis - sphenoid
- Sore throat
- Sputum
- Throat - pain
- Throat infection
- Tonsillar abscess
- Tonsillar concretion(s)
- Tonsillith(s)
- Tonsillitis
- Tonsillitis - bacterial
- Tonsillitis - chronic
- Tonsillitis - membranous
- Tonsillitis - recurrent
- Tracheitis
- Upper Airway Cough Syndrome
- Upper respiratory congestion
- Upper respiratory tract infection
- Upper respiratory tract infection - Recurrent
- Upper Respiratory Tract Infection - Virus
- URTI
- URTI - Bacterial
- URTI - recurrent
- URTI - Viral
- URTI - Viral with wheeze
- Vestibular neuronitis
- Vestibulitis - nasal
- Viral bronchitis
- Viral infection
- Viral labyrinthitis
- Viral pharyngitis
- Viral RTI
- Viral Upper Respiratory Tract Infection
- Viral URTI
- Virus Infection

After reviewing the data and consultation with clinical experts, any reason for visit including the following terms were excluded to refine the definition of URTI:

- “allergic”
- “smokers”
- “ACE inhibitor”
- “asthma”
- “non specific”
- “urticaria”
- “immunisation”
- “syncope”
- “gum”
- “pulmonary disease”
- “hoarseness”
- “salivary”
- “tonsillectomy”
- “wisdom tooth”
- Terms relating to ear infection (e.g., otalgia)
- Terms related to rhinitis or cough not caused by URTI (e.g., vasomotor rhinitis)
