# Supplementary material for: Burden of antimicrobial prescribing in primary care attributable to sore throat: a retrospective cohort study of patient record data
Source: BMC Prim Care. 2024 Apr 17;25:117. doi: 10.1186/s12875-024-02371-y (PMC11022400; doi:10.1186/s12875-024-02371-y)
Supplement: Supplementary file 2 — Supplementary Material 2. [file 12875_2024_2371_MOESM2_ESM.docx]

# Additional file 2

Antibiotics, and brand names, that are recommended for prescription for sore throat in Australia, as identified from the Australian Therapeutic Guidelines and with advice from clinical experts.

| Antibiotics | Brand Names |
| --- | --- |
| *Phenoxymethylpenicillin (Penicillin V)* | Aspecillin, Cilicaine V, Cilopen, LPV |
| *Benzylpenicillin (Penicillin G)* | BenPen, Bicillin, Cilicaine |
| *Cefalexin/Cephalexin* | Cephalex, Cephatrust, Cilex, Ialex, Ibilex, Keflex, Rancef |
| *Azithromycin* | Azith, Zedd, Zithro, Zithromax, Zitrocin |
| *Amoxicillin* | Alphamox, Amoxil, Amoxycillin, Bgramin, Cilamox, Fisamox, Ibiamox, Maxamox, Ranmoxy, Xycilan, Yomax |
| *Amoxicillin clavulanic-acid* | Alphaclav, Amclav, Amclavox, Amoxiclav, Amoxyclav, Augmentin, Augmentin Duo Forte, Clamoxyl Duo Forte, Clavam, Clavulin Duo Forte, Curam, Moxiclav |
| *Cefaclor* | Aclor, Ceclor, Karlor, Keflor, Ozcef |
| *Doxycycline* | Doryx, Doxsig, Doxy, Doxylin, Frakas |
| *Roxithromycin* | Biaxsig, Roxar, Roximycin, Rulide |
